# In reply: Comment on “Intraoperative superb microvascular ultrasound imaging in glioma: novel quantitative analysis correlates with tumor grade”

**DOI:** 10.1007/s00701-025-06585-6

**Published:** 2025-06-19

**Authors:** Matteo Palermo, Francesco Dimeco, Francesco Prada

**Affiliations:** 1 Department of Neurosurgery, Fondazione IRCCS Istituto Neurologico Carlo Besta, Via Celoria 11, 20133 Milan, Italy; 2 Department of Neurosurgery, Fondazione Policlinico Universitario A. Gemelli IRCCS, Università Cattolica del Sacro Cuore, Rome, Italy; 3 Department of Neurosurgery, Johns Hopkins University, Baltimore, MD USA; 4 Department of Neurological Surgery, University of Virginia Health System, Charlottesville, VA USA; 5 Focused Ultrasound Foundation, Charlottesville, VA USA

Dear Editor,

 I read with great interest the recent publication by Dixon et al., which proposes a novel quantitative analysis correlating microvascular patterns with tumor grade using intraoperative superb microvascular imaging (SMI) during glioma surgery [1]. However, while this contribution to advancing intraoperative ultrasound techniques in neuro-oncology is a milestone, it is worth noting that similar results have already been previously achieved by using contrast-enhanced ultrasound (CEUS) [2, 7, 9].

In 2014, Prada and colleagues published two landmark studies illustrating how intraoperative CEUS enabled real-time, dynamic imaging of the vascular structure of gliomas [5, 6]. This allowed for a quantitative evaluation of enhancement metrics, which effectively differentiated between high-grade and low-grade gliomas. A subsequent comparative study in 2017 confirmed the utility of CEUS when integrated with intraoperative MRI fusion imaging, guiding the intraoperative decision-making by comparing tumor enhancement patterns and margins, while still offering the advantages of portability, real-time acquisition, and the ability to be repeatedly used throughout surgery [5]. Moreover, in 2019 Kearns et al. reviewed the expanding role of CEUS in neurosurgical disease, highlighting its capacity to enhance intraoperative decision-making and reduce residual tumor rates [2]. This groundbreaking research established a foundation for utilizing intraoperative ultrasound as a means for functional tumor characterization, extending its traditional role in anatomical navigation.

Furthermore, in the last decade, CEUS has since expanded its applications within neurosurgical oncology. Beyond the mere application in tumor grading, CEUS has broadened its application to assessing intraoperative vascular complications, evaluating treatment response, and serving as an intraoperative surrogate for Gadolinium-enhanced MRI [6, 8]. For example, a recent study by Prada et al. (2021) carried an advanced quantitative analysis of in-vivo microbubble distribution in human brain tissue, opening new opportunities for intraoperative perfusion assessment and therapeutic monitoring [6]. The versatility and safety of CEUS, validated by the EFSUMB guidelines for non-hepatic applications, support its ever-since expanding adoption in neurosurgical practice [8].

Therefore, despite the study’s emphasis on the advantages of SMI as a contrast-free technique capable of delineating microvascular flow with impressive spatial resolution, CEUS remains unique in providing dynamic, real-time perfusion-based imaging. Omitting mention of CEUS as the pivotal modality in intraoperative settings for micro-vascular visualization represents a significant gap in the context of this work.

This is because contrast-enhanced techniques (CEUS or MRI) enable a level of physiological assessment that grayscale and Doppler-based techniques, including SMI, cannot fully replicate [3, 4]. Therefore, the lack of mention of CEUS, diverts attention from the wider discussion of the role of contrast agents in optimizing intraoperative imaging workflows. Rather than viewing these techniques as alternatives, we advocate for a complementary imaging approach.

Therefore, rather than viewing these techniques as alternatives, we advocate for a complementary imaging approach. It is believed that by integrating both contrast-enhanced and advanced Doppler-based modalities, surgeons can achieve a comprehensive intraoperative assessment, facilitating multiparametric analysis with pre-operative images [5]. Therefore, we would underline the importance of mentioning CEUS in this context, not only for its continued development and but also for the enhanced images that it is able to provide.

In conclusion, while the use of SMI represents a significant contribution to the field, its use should only be complementary rather than advocating for replacement of contrast-enhanced techniques. Future integrative studies combining SMI and CEUS could provide synergistic benefits for intraoperative imaging and further improve patient outcomes.

We commend the authors for their contribution to intraoperative ultrasound research and hope this letter highlights the valuable, established role of CEUS in neurosurgical oncology.

## Data Availability

No datasets were generated or analysed during the current study.

## Abbreviations

SMI: Superb Microvascular Imaging
CEUS: Contrast-Enhanced Ultrasound
MRI: Magnetic Resonance Imaging
EFSUMB: European Federation of Societies for Ultrasound in Medicine and Biology
